# GA/HbA1c ratio is a simple and practical indicator to evaluate the risk of metabolic dysfunction-associated fatty liver disease in type 2 diabetes: an observational study

**DOI:** 10.1186/s13098-022-00946-2

**Published:** 2022-11-11

**Authors:** Jun-Wei Wang, Chun-Hua Jin, Jiang-Feng Ke, Yi-Lin Ma, Yu-Jie Wang, Jun-Xi Lu, Mei-Fang Li, Lian-Xi Li

**Affiliations:** 1 Department of Endocrinology and Metabolism, Shanghai Sixth People’s Hospital Affiliated to Shanghai Jiao Tong University School of Medicine, Shanghai Clinical Center for Diabetes, Shanghai Diabetes Institute, Shanghai Key Laboratory of Diabetes Mellitus, Shanghai Key Clinical Center for Metabolic Disease, 600 Yishan Road, Shanghai, 200233 China; 2 Department of Endocrinology and Metabolism, Shanghai Songjiang District Central Hospital, Songjiang Hospital Affiliated to Shanghai Jiaotong University School of Medicine, Shanghai, 201600 China; 3 Department of Emergency, Shanghai Sixth People’s Hospital Affifiliated to Shanghai Jiao Tong University School of Medicine, 600 Yishan Road, Shanghai, 200233 China

**Keywords:** Glycated albumin/glycated hemoglobin A1C, GA/HbA1C ratio, Metabolic dysfunction-associated fatty liver disease, Non-alcoholic fatty liver disease, Insulin resistance, Type 2 diabetes mellitus

## Abstract

**Background:**

It is still debatable whether glycated albumin/glycated hemoglobin A1C (GA/HbA1C) ratio is associated with metabolic dysfunction-associated fatty liver disease (MAFLD), and few studies have been conducted in type 2 diabetes mellitus (T2DM). Therefore, we aimed to investigate the association between GA/HbA1C ratio and MAFLD and to evaluate whether GA/HbA1C ratio can be used an indicator of MAFLD in Chinese patients with T2DM.

**Methods:**

This cross-sectional study consisted of 7117 T2DM patients including 3296 men and 3821 women from real-world settings. Abdominal ultrasonography was performed to diagnose MAFLD. In addition to comparing the clinical characteristics among the GA/HbA1C ratio quartile groups, we also investigated the associations of GA/HbA1C ratio and quartiles with MAFLD in T2DM subjects.

**Results:**

There was a significantly decreased trend in the MAFLD prevalence across the GA/HbA1C ratio quartiles (56.3%, 47.4%, 37.8%, and 35.6% for the first, second, third, and fourth quartile, respectively, P < 0.001 for trend) after adjusting for gender, age, and diabetes duration. Fully adjusted Binary logistic regression indicated that both GA/HbA1C ratio (OR: 0.575, 95% CI: 0.471 to 0.702, P < 0.001) and quartiles (P < 0.001 for trend) were inversely associated with the presence of MAFLD among T2DM patients. Additionally, HOMA2-IR values were clearly increased in the T2DM subjects with MAFLD compared with those without MAFLD (P < 0.001), and markedly increased from the highest to the lowest GA/HbA1C ratio quartile (P < 0.001 for trend).

**Conclusions:**

GA/HbA1C ratio is closely and negatively associated with MAFLD in T2DM subjects, which may attribute to that GA/HbA1C ratio reflects the degree of insulin resistance. GA/HbA1C ratio may act as a simple and practical indicator to evaluate the risk of MAFLD in T2DM.

## Introduction

It is well established that non-enzymatic glycosylation of proteins are increased in patients with diabetes mellitus (DM) compared with non-diabetic subjects. Among these glycated proteins, glycated albumin (GA) reflects the short-term glycemic control over 2–3 weeks, while glycated hemoglobin (HbA1C) reflects the glycemic control status of the previous 2–3 months, which is widely used as the gold standard of glycemic control in clinical setting [1, 2]. On the other hand, glycated albumin/glycated hemoglobin A1C (GA/HbA1C) ratio was first described as an indicator to predict the future changes of HbA1C based on the fact that the changes in GA would precede those of HbA1C in type 2 diabetes mellitus (T2DM) patients [3]. In comparison with GA and HbA1C, GA/HbA1C ratio, with a half-life of 9 days, is a more reliable indicator of glycemic control and fluctuation within 2 weeks [4–6].

Therefore, GA/HbA1C ratio was reported to be linked to diabetic complications, cognitive impairment, and metabolic dysfunction [6–9], which were closely associated with poor glycemic control and increased glycemic variability. For example, several studies found a positive association between GA/HbA1C ratio and the prevalence and severity of diabetic complications such as carotid atherosclerosis, diabetic kidney disease, and retinopathy in T2DM subjects [8, 10, 11]. Moreover, previous studies noted that GA/HbA1C ratio was also closely related to metabolic disorders and risk factors such as abdominal obesity, non-alcoholic fatty liver disease (NAFLD), insulin resistance, and dyslipidemia [4, 12–14]. Of these metabolic disorders, NAFLD is considered as a liver manifestation of metabolic dysfunction in T2DM patients. Moreover, NAFLD is approximately twofold more prevalent in patients with T2DM compared to those without diabetes [15–17]. According to the new definition of NAFLD, metabolic dysfunction-associated fatty liver disease (MAFLD) typically involves not only hepatic steatosis, but also obesity, T2DM, and other metabolism-related risk factors [15]. At present, although there are studies on the link between GA/HbA1C ratio and liver damage, the results are still controversial. Furthermore, studies focused on the association between GA/HbA1C ratio and MAFLD are lacking. For example, several studies demonstrated that the increased GA/HbA1C ratio is significantly associated with liver fibrosis progresses in patients with chronic hepatitis [18–20]. However, another study supposed that low but not high GA/HbA1C ratio might result in increased hepatic inflammation in patients with chronic liver disease [21].

Likewise, the relationship between GA/HbA1C ratio and hepatic injury is also controversial in patients with T2DM, and few studies investigate the relevance between GA/HbA1C ratio and MAFLD. A previous study verified an increased prevalence of NAFLD with the decrease of GA/HbA1C ratio in patients with T2DM [4]. However, after full adjustment for confounders, the moderate GA/HbA1C ratio did not increased the prevalence of NAFLD compared to the highest GA/HbA1C ratio in T2DM patients [4]. Interestingly, a previous study suggested that the GA/HbA1C ratio was higher in patients with chronic liver disease than in T2DM patients without chronic liver disease [20], which indicates the GA/HbA1C ratio may be linked with hepatic function but not with the levels of blood glucose.

Therefore, a lack of studies exists in the relationship between GA/HbA1C ratio and MAFLD in T2DM patients. The aims of the present study are to investigate the correlation between GA/HbA1C ratio and MAFLD and to explore relevant factors in Chinese T2DM subjects.

## Materials and methods

### Subjects and study design

The T2DM patients in this cross-sectional, real-world study were recruited from the Endocrinology and Metabolism Department, Shanghai Sixth People’s Hospital Affifiliated to Shanghai Jiao Tong University School of Medicine spanning January 2003–December 2012. Besides, some data came from our recent studies [22–24]. Upon receiving written consent from all participants, this study was approved by the ethical review committee of the hospital (approved number: 2018-KY-018(K))**.** The criteria for exclusion were as follows: incomplete clinical data; liver diseases caused by drugs, virus and other reasons excluding alcohol; disorders influencing hemoglobin and albumin metabolism such as anemia and hyperthyroidism; severe systemic diseases or infectious diseases. Based on GA/HbA1C ratio, 7117 T2DM patients were ultimately recruited and divided into four quartile groups in the present study.

### Physical examination and laboratory tests

According to our previous description, the following information was collected at admission: history of hypertension, diabetes duration (DD), alcohol consumption, smoking habits, administration of lipid-lowering drugs (LLDs), metformin, insulin sensitizers, and insulin or insulin analogs (IIAs) [22, 23, 25, 26]. Body measurements including height, waist and hip circumference, weight, and blood pressure were taken during the physical examination at admission. The definitions of hypertension, obesity, smoking and alcohol status were detailedly described in our previous studies [22, 23, 25]. Likewise, waist-to-hip ratio (WHR) was calculated as waist circumstances (WC) divided by hip circumstances, and body mass index (BMI) was determined as weight divided by height squared as previously described [22, 23, 25].

In the morning of the second day following admission, blood samples were taken after fasting overnight and two hours after breakfast. Serum HbA1C was determined using high-performance liquid chromatography. Serum GA, alanine transaminase (ALT) and gamma-glutamyl transferase (γ-GT) were measured by enzymatic method. The homeostasis model assessment of insulin resistance (HOMA2-IR) and insulin sensitivity (HOMA2-S), the estimated glomerular filtration rate (eGFR) and laboratory parameters including blood glucose, insulin, C-peptide, lipids, urine tests and kidney function were determined and calculated according to our previous studies [22–26].

### Abdominal ultrasonography and diagnosis of MAFLD

Abdominal ultrasound examinations and diagnosis of hepatic steatosis conformed to our previous studies [25, 26]. Given that all subjects were T2DM patients in this study, we identified MAFLD through ultrasonographic confirmation of hepatic steatosis as well as the presence of T2DM, which was developed by an expert panel from twenty-two different countries [15]**.**

### Statistical analysis

Data were analyzed with SPSS 15.0. For continuous variables, normal distribution was assessed and then data were expressed as mean ± standard deviation or median and interquartile range. To evaluate the differences between the two groups, t-tests or Mann–Whitney U tests were employed. The differences across multiple groups were evaluated using one-way ANOVA or Kruskal–Wallis H tests. Chi-square tests were used to analyze categorical variables. Categorical variables were controlled with binary logistic regression, and continuous variables were adjusted with univariate linear regression when adjusting for gender and age. Binary logistic regression was applied to examine the association of GA, HbA1C, and GA/HbA1C ratio and quartiles with the MAFLD presence. This difference was statistically significant at P < 0.05.

## Results

### Characteristics of the study subjects

This study included 7117 inpatients with T2DM. In accordance with GA/HbA1C ratio quartiles with cutoffs of < 2.45, 2.45–2.72, 2.73–3.16, and > 3.16, the study subjects were classified into four groups. Table 1 compares the baseline characteristics of the subjects among the four groups. Following adjustment for sex and age, the prevalence of hypertension and obesity, smoking status, metformin and LLDs usage, WC, BMI, and levels of low-density lipoprotein cholesterol (LDL-C) and serum uric acid (SUA) were found to significantly decreased along with fasting plasma glucose (FPG), 2-h postprandial plasma glucose (2-h PPG), and GA values obviously increased from the lowest to highest GA/HbA1C ratio quartile (all P < 0.05). In addition, there were significant difference in age, gender, DD, alcohol intake, insulin sensitizers and IIAs usage, WHR, systolic blood pressure (SBP), HbA1C, percentage of patients with HbA1C < 7%, albumin, fasting C-peptide (FCP), 2-h postprandial C-peptide (2 h C-P), total triglycerides (TG), total cholesterol (TC), high-density lipoprotein cholesterol (HDL-C), urinary albumin excretion (UAE), and C-reactive protein (CRP) values among the four groups (all P < 0.05). However, there were no significant difference in diastolic blood pressure (DBP), creatinine (Cr), or eGFR among the GA/HbA1C ratio quartiles.

**Table 1:** Characteristics of the subjects according to GA/HbA1C ratio

Variables	Q1 (n = 1776)	Q2 (n = 1798)	Q3 (n = 1803)	Q4 (n = 1740)	P value	*P value
GA/HbA1c	< 2.45	2.45–2.72	2.73–3.16	> 3.16	–	–
Male (n, %)	754 (42.5%)	824 (45.8%)	867 (48.1%)	851 (48.9%)	< 0.001	< 0.001
Age (years)	58 ± 13	60 ± 13	60 ± 13	62 ± 13	< 0.001	< 0.001
*DD (months)	96 (36–156)	96 (36–168)	108 (36–168)	84 (24–156)	< 0.001	< 0.001
Hypertension (n, %)	1016 (57.2%)	1005 (55.9%)	956 (53.0%)	918 (52.8%)	0.017	< 0.001
Obesity (n, %)	1066 (60.0%)	897 (49.9%)	704 (39.0%)	655 (37.6%)	< 0.001	< 0.001
Smoking (n, %)	393 (22.1%)	395 (22.0%)	358 (19.9%)	339 (19.5%)	0.104	< 0.001
Alcohol (n, %)	305 (17.2%)	280 (15.6%)	326 (18.1%)	268 (15.4%)	0.050	0.045
IIAs (n, %)	1201 (67.6%)	1195 (66.5%)	1346 (74.7%)	1299 (74.7%)	< 0.001	< 0.001
LLDs (n, %)	880 (49.5%)	760 (42.3%)	634 (35.2%)	507 (29.1%)	< 0.001	< 0.001
Metformin (n, %)	1192 (67.1%)	1130 (62.8%)	1006 (55.8%)	887 (51.0%)	< 0.001	< 0.001
Insulin sensitizers (n,%)	309 (17.4%)	250 (13.9%)	271 (15.0%)	153 (8.8%)	< 0.001	< 0.001
SBP (mmHg)	133 ± 17	132 ± 17	132 ± 18	132 ± 18	0.676	0.04
DBP (mmHg)	81 ± 9	80 ± 10	80 ± 9	80 ± 10	0.008	0.089
WC (cm)	92.05 ± 10.39	90.34 ± 10.23	88.02 ± 10.04	86.90 ± 10.44	< 0.001	< 0.001
WHR	0.93 ± 0.06	0.92 ± 0.07	0.91 ± 0.07	0.91 ± 0.07	< 0.001	< 0.001
BMI (kg/m2)	26.04 ± 3.58	25.35 ± 3.44	24.35 ± 3.28	24.02 ± 3.42	< 0.001	< 0.001
*FPG(mmol/l)	7.41 (6.01–9.30)	7.51(6.03–9.39)	7.87(6.26–10.02)	8.15 (6.50–10.49)	< 0.001	< 0.001
*2 h PPG(mmol/l)	12.49 (9.61–15.74)	13.23(9.93–16.51)	13.76(10.68–17.21)	14.26 (11.00–17.80)	< 0.001	< 0.001
HbA1C (%)	8.95 ± 2.20	8.77 ± 2.22	9.13 ± 2.32	9.14 ± 2.36	< 0.001	< 0.001
HbA1C < 7% (n, %)	347 (19.5%)	433(24.1%)	373(20.1%)	333 (19.1%)	0.001	0.001
GA(%)	19.00 (16.20–23.00)	21.50(18.00–26.00)	26.00(21.00–31.00)	43.00 (30.00–330.83)	< 0.001	< 0.001
*FCP (ng/mL)	2.05 (1.38–2.87)	1.87(1.26–2.64)	1.62(1.04–2.36)	1.67 (0.98–2.54)	< 0.001	< 0.001
*2 h C-P (ng/mL)	4.63 (2.75–6.74)	4.37(2.62–6.25)	3.42(2.07–5.53)	3.71 (1.95–5.87)	< 0.001	< 0.001
*TG (mmol/l)	1.62 (1.14–2.41)	1.46(1.03–2.14)	1.33(0.92–1.96)	1.33 (0.93–1.98)	< 0.001	< 0.001
TC (mmol/l)	4.90 ± 1.20	4.78 ± 1.13	4.73 ± 1.15	4.78 ± 1.13	< 0.001	0.008
HDL-C (mmol/l)	1.10 ± 0.29	1.11 ± 0.28	1.16 ± 0.33	1.16 ± 0.33	< 0.001	< 0.001
LDL-C (mmol/l)	3.12 ± 0.99	3.06 ± 0.95	3.02 ± 0.93	3.01 ± 0.90	0.001	0.018
Albumin (g/l)	42.7 ± 4.12	43.2 ± 3.55	43.2 ± 3.65	41.9 ± 4.00	< 0.001	0.014
*Cr (μmol/l)	63.0 (52.0–76.0)	65.0 (54.0–77.0)	65.0 (54.0–79.0)	67.0 (55.0–80.0)	< 0.001	0.122
*SUA (μmol/l)	324 (272–385)	312 (256–371)	300 (245–363)	298 (248–360)	< 0.001	< 0.001
*UAE (mg/24 h)	13.99 (7.34–58.51)	11.53 (6.85–31.96)	11.03 (6.65–24.45)	11.80 (6.99–28.46)	< 0.001	< 0.001
*eGFR (ml/min/1.73 m^2^)	114.0 (93.4–136.9)	109.9 (89.4–134.7)	109.9 (91.5–134.1)	106.5 (87.1–128.6)	< 0.001	0.18
*CRP (mg/l)	1.36 (0.63–3.14)	1.11 (0.50–2.69)	1.03 (0.45–2.57)	1.20 (0.51–3.42)	< 0.001	< 0.001

Values are expressed as the mean ± S.D, or median with interquartile range, or percentages

p value: the p-values were not adjusted for age and sex for the trend

p* value: the p-values were adjusted for sex and age for the trend

^*^The Kruskal–Wallis test was applied

### Comparisons of MAFLD prevalence and GA/HbA1C ratio stratified by sex, age, and DD

**Fig. 1:**
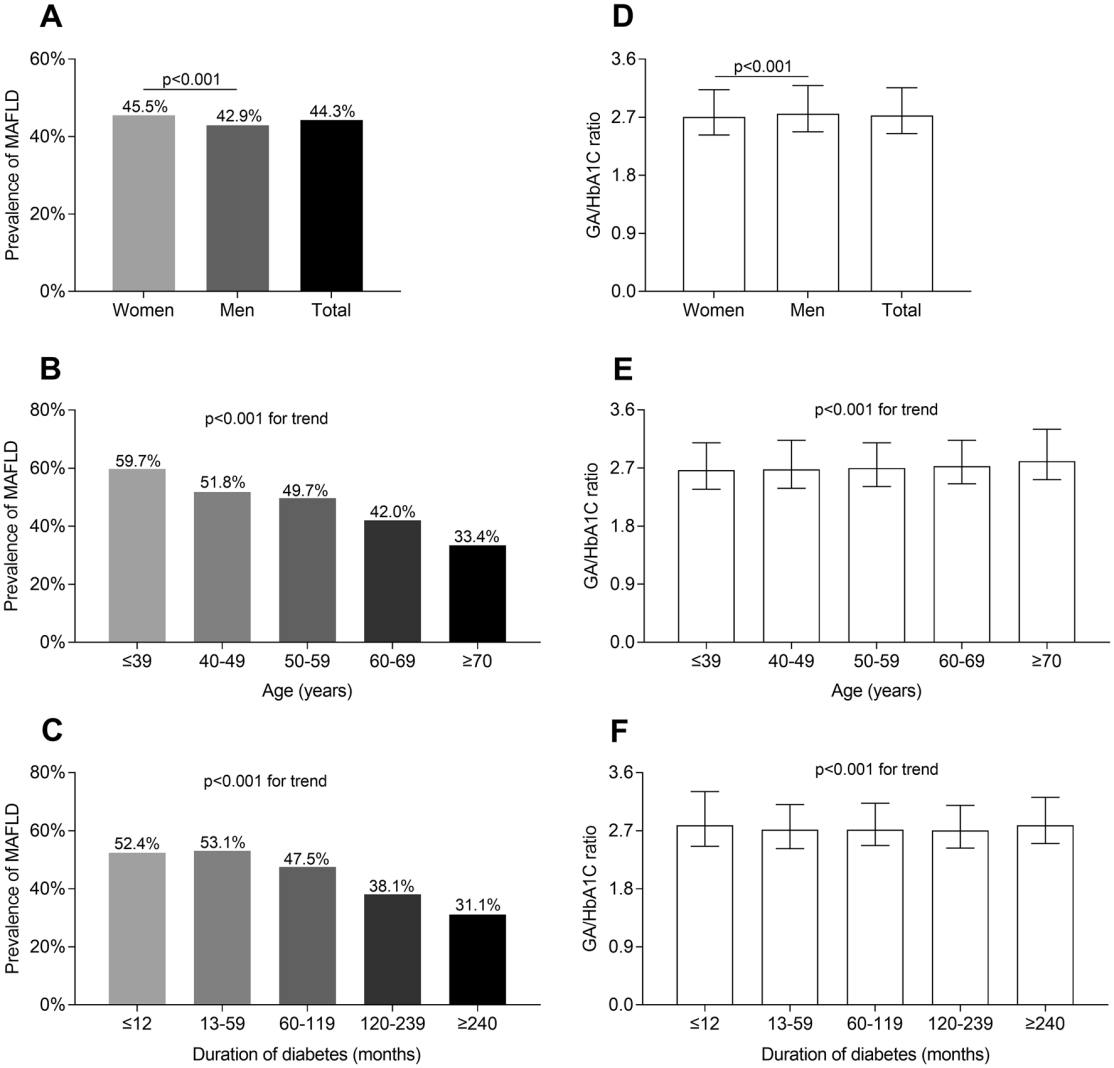
Comparisons of MAFLD prevalence and GA/HbA1C ratio stratified by sex, age, and DD. **A** Overall prevalence of MAFLD and comparisons of the MAFLD prevalence stratified by gender (P < 0.001). **B** Comparisons of the MAFLD prevalence stratified by age (P < 0.001 for trend). **C** Comparisons of the MAFLD prevalence stratified by DD (P < 0.001 for trend). **D** Overall GA/HbA1C ratio and comparisons of GA/HbA1C ratio stratified by gender (P < 0.001). **E** Comparisons of GA/HbA1C ratio stratified by age (P < 0.001 for trend). **F** Comparisons of GA/HbA1C ratio stratified by DD (P < 0.001 for trend)

The comparisons of MAFLD prevalence stratified by gender, age, and DD are presented in Fig. 1. The prevalence of MAFLD were found to be higher in women than in men (P < 0.001, Fig. 1A). Further, it was evident that the prevalence of MAFLD significantly decreased in line with aging and extended DD (both P < 0.001 for trend) (Fig. 1B and C). Moreover, there were lower GA/HbA1C ratio in women compared to men (P < 0.001, Fig. 1D). In addition, GA/HbA1C ratio increased with increasing age (P < 0.001 for trend) but decreased with prolonged DD (P < 0.001 for trend) (Fig. 1E and F).

### Comparisons of GA/HbA1C ratio and MAFLD prevalence

**Fig. 2:**
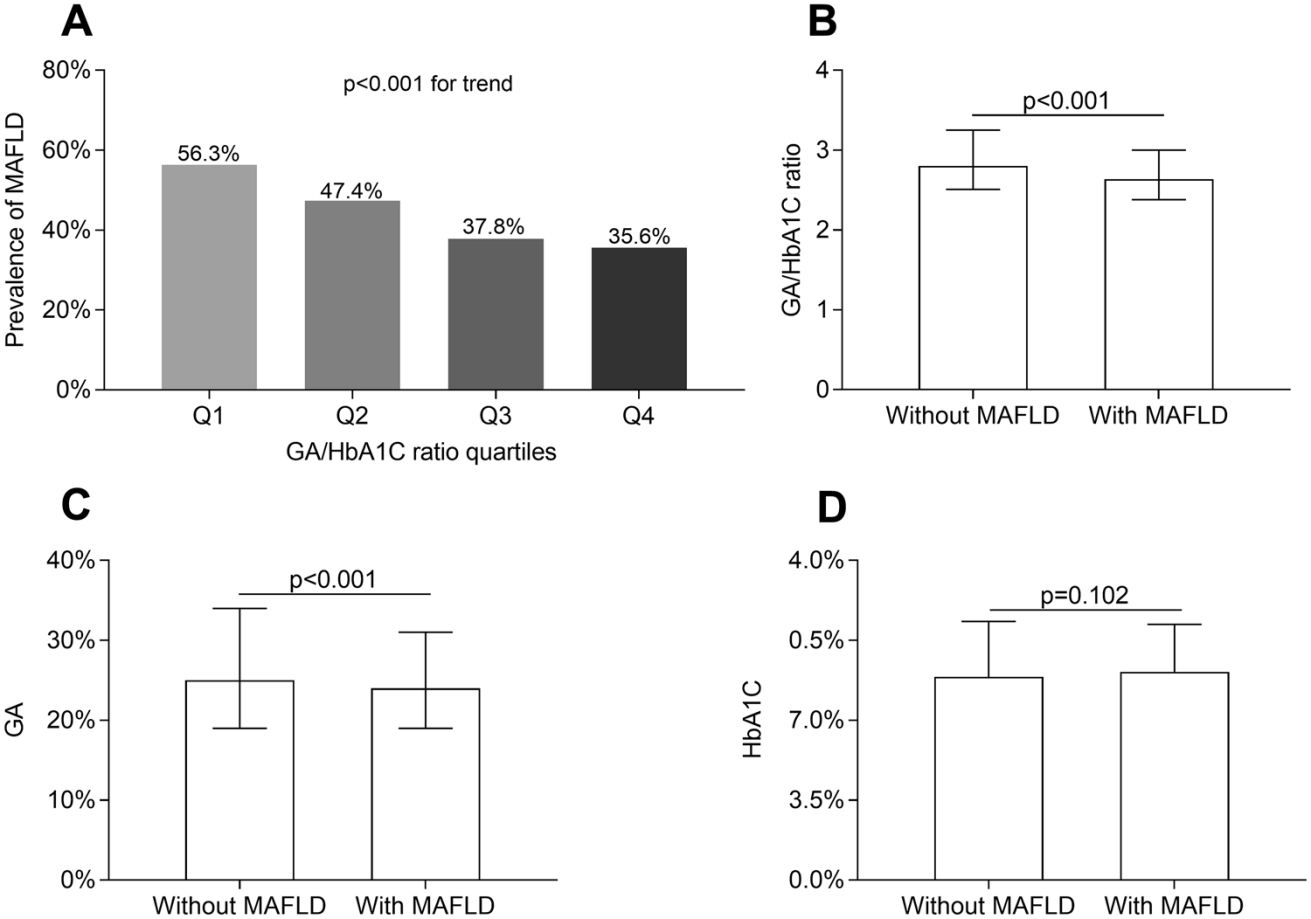
Comparisons of GA, HbA1C, and GA/HbA1C ratio. **A** Comparisons of the MAFLD prevalence across the GA/HbA1C ratio groups (P < 0.001 for trend). **B** Comparisons of GA/HbA1C ratio between the patients with and without MAFLD (P < 0.001). **C** Comparisons of GA between the patients with and without MAFLD (P < 0.001). **D** Comparisons of HbA1C between the patients with and without MAFLD (P < 0.001)

Figure 2 compares the prevalence of MAFLD among the GA/HbA1C ratio quartiles and the values of GA/HbA1C ratio between the T2DM patients with and without MAFLD. There was a significantly decreased trend in the MAFLD prevalence across the GA/HbA1C ratio quartiles after adjusting for age, gender, and DD (56.3%, 47.4%, 37.8%, and 35.6% for the first, second, third, and fourth quartile, respectively, P < 0.001 for trend) (Fig. 2A). In addition, the values of GA/HbA1C ratio and GA in the T2DM patients with MAFLD were distinctly lower than in those without MAFLD (all P < 0.001) (Fig. 2B and Fig. 2C). However, there was no difference in HbA1C levels between the T2DM patients with and without MAFLD (P = 0.102) (Fig. 2D).

### Comparisons of ALT and γ-GT

**Fig. 3:**
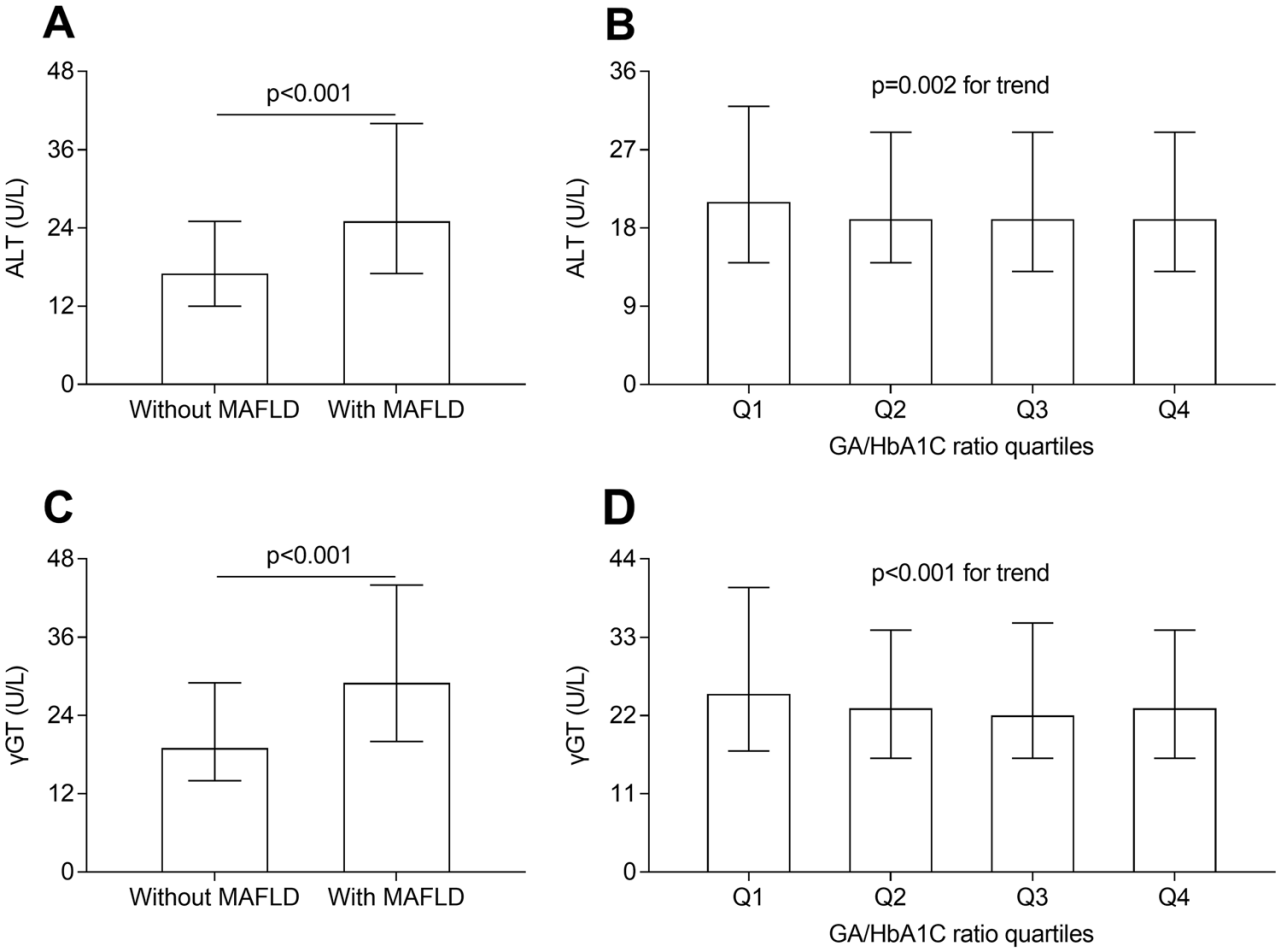
Comparisons of serum ALT and γ-GT levels. **A** Comparison of serum ALT levels between the patients with and without MAFLD (P < 0.001). **B** Comparison of serum ALT levels across the GA/HbA1C ratio quartile groups (P = 0.002 for trend). **C** Comparison of serum γ-GT levels between the patients with and without MAFLD (P < 0.001). **D** Comparison of serum γ-GT levels across the GA/HbA1C ratio quartile groups (P < 0.001 for trend)

Figure 3 illustrates the comparisons of serum ALT and γ-GT among different groups. After correction for age, sex, and DD, serum ALT and γ-GT values were clearly higher in the patients with MAFLD than in those without MAFLD (both P < 0.001, Fig. 3A and C). Moreover, both ALT and γ-GT levels were significantly lower in the second, third, and fourth quartile compared to the first quartile (P = 0.002 for trend, Fig. 3B and P < 0.001 for trend, Fig. 3D).

### Comparisons of HOMA2-IR and HOMA2-S

**Fig. 4:**
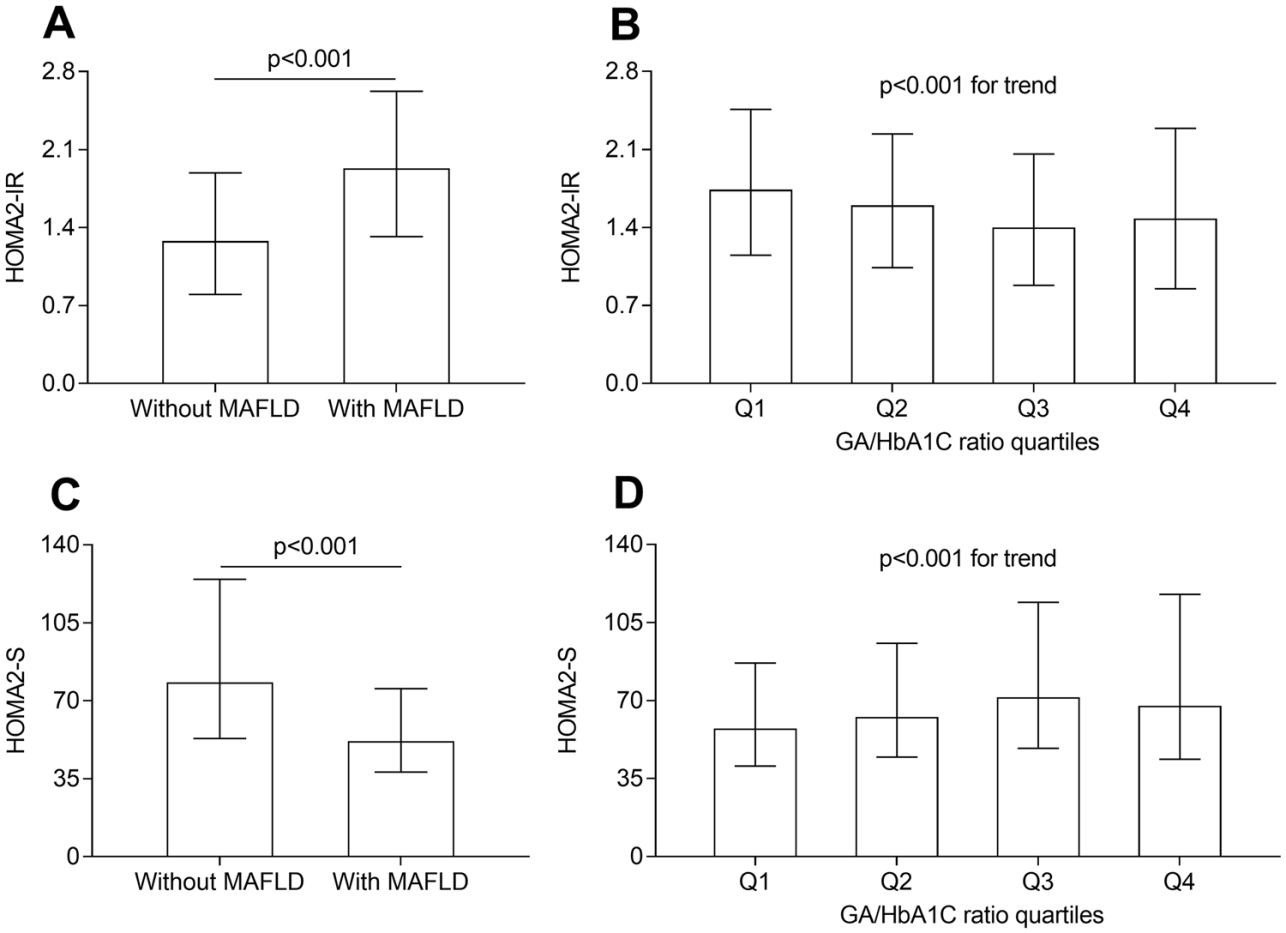
Comparisons of HOMA2-IR and HOMA2-S. **A** Comparison of HOMA2-IR between the patients with and without MAFLD (P < 0.001). **B** Comparison of HOMA2-IR across the GA/HbA1C ratio quartile groups (P < 0.001  for trend). **C** Comparison of HOMA2-S between the patients with and without MAFLD (P < 0.001). **D** Comparison of HOMA2-S across the GA/HbA1C ratio quartile groups (P < 0.001 for trend)

Figure 4 compares HOMA2-IR and HOMA2-S between the T2DM patients with and without MAFLD, and correspondingly among GA/HbA1C ratio quartiles. As adjusted for sex, age, and DD, HOMA2-IR was significantly higher and HOMA2-S was obviously lower in the T2DM patients with MAFLD compared to those without MAFLD (all P < 0.001, Fig. 4A and C). Further comparisons showed a significantly decreased trend in HOMA2-IR (P < 0.001 for trend, Fig. 4B), as well as a clearly increased trend in HOMA2-S (P < 0.001 for trend, Fig. 4D) across GA/HbA1C ratio quartiles.

### Association of GA/HbA1C ratio with MAFLD

In Table 2, Binary logistic regression was conducted to explore the association between GA/HbA1C ratio and the presence of MAFLD. In Model 1 without adjustment for confounders, decreased GA/HbA1C ratio was associated with increased risk for MAFLD prevalence (P < 0.001). After further controlling for confounding factors, a significant negative association between GA/HbA1C ratio and MAFLD was still exist (P < 0.001 in Model 2, 3, 4, and 5). Moreover, even after controlling for GA and HbA1C, a low GA/HbA1C ratio was also stably and closely associated with an increased prevalence of MAFLD (P < 0.001 in Model 6).

**Table 2:** Association of GA/HbA1c ratio with MAFLD

	B statistic	OR	95% CI	*P* value
Model 1	−0.306	0.736	0.702–0.773	< 0.001
Model 2	−0.190	0.827	0.784–0.872	< 0.001
Model 3	−0.139	0.871	0.825–0.919	< 0.001
Model 4	−0.173	0.841	0.783–0.904	< 0.001
Model 5	−0.162	0.851	0.781–0.926	< 0.001
Model 6	−0.553	0.575	0.471–0.702	< 0.001

Model 1: unadjusted

Model 2: adjusted for age, sex, DD, smoking status, alcohol intake, obesity, and hypertension

Model 3: further adjustment for use of LLD, IIAs, metformin, and insulin sensitizers

Model 4: further adjustment for SBP, DBP, WC, WHR, and BMI

Model 5: further adjustment for TC, TG, HDL-C, LDL-C, eGFR, Cr, SUA, UAE, FCP, 2-h CP, HOMA2-IR, FPG, 2-h PPG, and CRP

Model 6: further adjustment for GA and HbA1C

### Association of GA/HbA1C ratio quartiles with MAFLD

A detailed analysis of the GA/HbA1C ratio quartiles in association with MAFLD in T2DM subjects is presented in Table 3. The GA/HbA1C ratio quartiles were negatively correlated with the presence of MAFLD without controlling for confounding factors (P < 0.001 for trend, Model 1). Then, despite adjusting for sex, age, and DD, smoking, alcohol status, hypertension, and obesity (Model 2), therapy for diabetes and hyperlipidemia (Model 3), parameters of physical examination (Model 4), and laboratory parameters (Model 5), GA/HbA1C ratio quartiles were still inversely associated with the MAFLD presence (P < 0.001 for trend in Model 2, 3, 4, and P = 0.005 for trend in Model 5). Even after further adjustment for GA and HbA1C, higher GA/HbA1C ratio quartiles were still independently associated with lower risk of MAFLD in T2DM patients (P < 0.001 for trend, Model 6).

**Table 3:** Association of GA/HbA1C ratio quartiles with MAFLD

	ORs (95% CI)				P values for trend
	Q1	Q2	Q3	Q4	
Model 1	1	0.700 (0.614–0.799)	0.473 (0.414–0.541)	0.429 (0.375–0.492)	< 0.001
Model 2	1	0.836 (0.724–0.966)	0.639 (0.552–0.741)	0.570 (0.490–0.662)	< 0.001
Model 3	1	0.873 (0.754–1.011)	0.698 (0.601–0.811)	0.654 (0.561–0.762)	< 0.001
Model 4	1	0.878 (0.751–1.025)	0.768 (0.655–0.899)	0.630 (0.520–0.764)	< 0.001
Model 5	1	0.860 (0.722–1.025)	0.772 (0.643–0.927)	0.690 (0.551–0.865)	0.005
Model 6	1	0.735 (0.611–0.885)	0.595 (0.484–0.731)	0.460 (0.352–0.602)	< 0.001

Model 1: unadjusted

Model 2: adjusted for age, sex, DD, smoking status, alcohol intake, obesity, and hypertension

Model 3: further adjustment for use of LLD, IIAs, metformin, and insulin sensitizers

Model 4: further adjustment for SBP, DBP, WC, WHR, and BMI

Model 5: further adjustment for TC, TG, HDL-C, LDL-C, eGFR, Cr, SUA, UAE, HbA1c, FCP, 2-h CP, HOMA2-IR, FPG, 2-h PPG, and CRP

Model 6: further adjustment for GA and HbA1C

## Discussion

GA/HbA1C ratio was first described in 1991 to predict the change in HbA1C in the forthcoming months[3]. It is now generally accepted that GA/HbA1C ratio reflects glucose fluctuation such as mean amplitude of glycemic excursions, irrespective of the type of diabetes[27]. Moreover, several studies supported a positive correlation between GA/HbA1C ratio and diabetic complications [10, 28, 29]. For example, a previous study found that increased GA/HbA1C ratio reflected more severe oxidative stress than chronic sustained hyperglycemia and was positively associated with diabetic kidney disease [29]. In addition, a related study underlined that GA/HbA1C ratio was linked with postprandial hyperglycemia and glucose excursion which promoted endothelial dysfunction, and the latter was a well-known factor for atherosclerosis [28]. Moreover, increased GA/HbA1C ratio was reported to reflect the aggravation of diabetic retinopathy, as it indicates increased early Amadori-type glycation product inducing inflammatory mediators in the vascular wall [10, 30]. In addition, a previous study suggested that higher GA levels were associated with all-cause and cardiovascular mortality [31]. Therefore, GA/HbA1C ratio might act as a predictor for diabetic chronic complications.

However, studies on the relationship between GA/HbA1C ratio and MAFLD in the general and diabetic populations have been rarely reported so far, and the overall results were inconclusive. Several previous studies supported the association between an elevated GA/HbA1C ratio and the increased prevalence and severity of liver fibrosis in patients with chronic hepatitis [18–20]. For example, Aizawa et al. found that patients with cirrhosis had a mean GA/HbA1c ratio of 3.14, which was significantly higher than GA/HbA1c ratio of 2.85 in those without cirrhosis [18]. However, a previous study found that low but not high GA/HbA1C ratio might be an indicator to assess the severity of liver injury in patients with chronic liver disease [20]. Therefore, the association between GA/HbA1C ratio and liver damage is still inconsistent, and no studies linking GA/HbA1C ratio to MAFLD exist in general population.

Similarly, given the few relevant investigations, the correlation of GA/HbA1C ratio with MAFLD was controversial in patients with T2DM. In a study of T2DM patients, it was noted that NAFLD patients had lower GA/HbA1C ratio compared with non-NAFLD patients [4]. However, another study conducted in T2DM patients indicated that there was a positive correlation between GA/HbA1C ratio and the progression of fibrosis in patients with NAFLD [32]. Therefore, we performed the present study with large samples to explore the association between GA/HbA1C ratio and MAFLD in T2DM subjects and found that 44.3% of T2DM patients had MAFLD, which was close to the NAFLD prevalence in our previous studies (39.4%-52.6%) [25, 33].

More importantly, we found that the increased GA/HbA1C ratio was closely linked with the decreased risk of MAFLD even after controlling for other confounding factors including GA and HbA1C. The risk of MAFLD reduced by 43% with each 1 SD increase in GA/HbA1C ratio. Consistent with our results, a previous investigation in a small sample of T2DM subjects showed that the lowest GA/HbA1c tertile was related to 2.75-fold higher risk for NAFLD comorbidity and 4.48-fold higher risk for NAFLD progression compared to the highest GA/HbA1c tertile [4]. Additionally, we observed the risk of MAFLD prevalence decreased by more than half when GA/HbA1C ratio was greater than 3.16. Moreover, the MAFLD prevalence was gradually decreased from the first to the fourth GA/HbA1C ratio quartile. Even though after adjusting for GA and HbA1C, decreased GA/HbA1C ratio was still an independent factor for the risk of MAFLD in T2DM patients.

Interestingly, the present study suggested a decreased tendency for serum ALT and γ-GT levels with the increase in GA/HbA1C ratio quartiles. Given the fact that serum ALT and γ-GT levels were positively correlated with the degree of fat infiltration in liver and NAFLD activity score [30], low GA/HbA1C ratio were also thought to reflect the degree and progression of MAFLD in T2DM patients in our study. Consistent with our findings, several studies also demonstrated that GA/HbA1C ratio was negatively correlated with ALT and γ-GT in patients with and without T2DM [4, 32, 34]. For example, a previous study including NAFLD patients with diabetes found that GA/HbA1C ratio had an inverse correlation with ALT and its coefficient was -0.572 [32]. Additionally, in another study also including T2DM patients, a mean value of serum ALT was 19U/L in low GA/HbA1C ratio group, which was higher compared to 17U/L in high GA/HbA1C ratio group [34]. Similarly, another T2DM population-based study also found that during the progression of NAFLD, serum ALT increased from 16U/L to 33U/L, as well as serum γ-GT rose from 22U/L to 49.5U/L accompanied with the decline of GA/HbA1C ratio [4]. Therefore, the reduced GA/HbA1C ratio might indicate the severity of hepatic injury and progression of fatty liver in T2DM subjects.

This negative association between GA/HbA1C ratio and MAFLD can be partially explained by the inverse correlation between GA/HbA1C ratio and insulin resistance. It is clear that insulin resistance is the main mechanism underlying the development of MAFLD, which stimulates the de novo synthesis of fatty acids and inhibits mitochondrial β-oxidation in hepatocytes, and then leads to the occurrence of MAFLD [35]. Consistently, a recent study among non-diabetic patients found a negative correlation between GA/HbA1C ratio and HOMA-IR [12]. Another study conducted in subjects with prediabetes also indicated that GA/HbA1C ratio was weakly but negatively associated with HOMA-IR with a coefficient of −0.138 [36]. Similarly, a previous study displayed that mean HOMA-IR decreased from 3.6 to 1.7 with an increase of GA/HbA1C ratio tertiles in T2DM patients [37]. We also found a trend towards a decrease in HOMA2-IR and an increase in HOMA2-S from low to high GA/HbA1C ratio quartiles. Therefore, increased insulin resistance linked to low GA/HbA1C ratio might contribute to the development and progression of MAFLD in T2DM.

In addition, we found that WC, BMI, and percentage of obesity decreased accompanied with increased GA/HbA1C ratio, which suggested a negative correlation between GA/HbA1C ratio and obesity. It was repeatedly confirmed that GA/HbA1C ratio was negatively correlated with obesity, and the latter was a well-established risk factor for MAFLD [9, 15, 38–40]. For example, a previous study including T2DM patients reported 37.4% of obesity in low GA/HbA1C ratio group compared to 19.2% of obesity in high GA/HbA1C ratio group [34]. Moreover, we found that increased percentage of hypertension from lower to higher GA/HbA1C ratio quartile groups, and a recent meta-analysis indicated that NAFLD was associated with 1.66-fold increased risk of developing hypertension [41]. Hence, the patients with reduced GA/HbA1C ratio were more prone to MAFLD in our study, which was partially explained by the negative correlation between obesity, hypertension and GA/HbA1C ratio.

Furthermore, the negative association between GA/HbA1C ratio and MAFLD prevalence might also be explained by CRP and serum albumin concentrations. We observed that decreased CRP levels in the second, third, and fourth GA/HbA1C ratio quartiles compared with the first quartile, which was consistent with a significant negative correlation between GA/HbA1C ratio and CRP by a previous study [21]. Additionally, serum albumin concentrations were lower in the fourth GA/HbA1C ratio quartile than in the first, second, and third ratio quartiles based on our results. Similarly, Bando et al. found a negative association between GA/HbA1C and albumin in patients with chronic liver disease [20]. As a result, lower GA/HbA1C ratio was accompanied with higher CRP and albumin levels, which might be linked to increased MAFLD prevalence.

Our study had some limitations. First, several factors were reported to be associated with levels of GA and HbA1C such as alcohol intake, smoking, lipid profile, and SUA [12–14]. However, we adjusted these confounding factors to reduce their effect on the results as much as possible. Second, since this was a cross-sectional study, we failed to identify a causal link between GA/HbA1C ratio and MAFLD prevalence. Therefore, a prospective cohort study would be needed to further clarify the association of GA/HbA1C ratio with MAFLD. However, the present study was based on a real-world background with a large sample size and fully adjustment of confounding factors, which supported relatively reliable conclusions. Third, milder MAFLD might be unnoticed by abdominal ultrasonography. Despite this, it remained a major diagnostic tool for MAFLD in large population, because it was noninvasive, inexpensive, time-saving and highly related to pathological measurement of fatty liver [42]. Fourth, there was a lack of quantitative data on MAFLD in our study, such as score of fibrosis and degree of steatosis assessing by liver transient elastography. However, it has been noted that serum ALT and γ-GT levels increased with the degree of fibrosis and NAFLD status determined by transient elastography [43–45], and our findings suggested that GA/HbA1C ratio was closely correlated with ALT and γ-GT, which were also indicators reflecting the severity of MAFLD.

## Conclusions

GA/HbA1C ratio is negatively associated with MAFLD independent of plasma glucose levels in T2DM patients, which may attribute to that GA/HbA1C ratio indicates the degree of insulin resistance. GA/HbA1C ratio may be used as a simple and practical indicator to evaluate the risk of MAFLD in T2DM.

## Data Availability

The datasets used and/or analyzed during the current study are available from the corresponding author on reasonable request.

## Abbreviations

HbA1C: Glycated hemoglobin A1C
GA: Glycated albumin
GA/HbA1C: Glycated albumin-to-glycated hemoglobin ratio
T2DM: Type 2 diabetes mellitus
NAFLD: Non-alcoholic fatty liver disease
MAFLD: Metabolic dysfunction-associated fatty liver disease
DD: Diabetes duration
SBP: Systolic blood pressure
DBP: Diastolic blood pressure
WC: Waist circumference
LLDS: Lipid-lowering drugs
BMI: Body mass index
IIAs: Insulin or insulin analogue
WHR: Waist-to-hip ratio
FPG: Fasting plasma glucose
2-h PPG: 2-H postprandial plasma glucose
2-h C-P: 2-H postprandial C-peptide
HOMA2-IR: HOMA of insulin resistance
HOMA2-S: HOMA of insulin sensitivity
TG: Total triglycerides
TC: Total cholesterol
FCP: Fasting C-peptide
HDL-C: High-density lipoprotein cholesterol
LDL-C: Low-density lipoprotein cholesterol
ALT: Alanine transaminase
Cr: Creatinine
SUA: Serum uric acid
UAE: Urinary albumin excretion
eGFR: Estimated glomerular filtration rate
CRP: C-reactive protein
